# How Should Staphylococcal Food Poisoning Outbreaks Be Characterized? 

**DOI:** 10.3390/toxins2082106

**Published:** 2010-08-10

**Authors:** Jacques-Antoine Hennekinne, Annick Ostyn, Florence Guillier, Sabine Herbin, Anne-Laure Prufer, Sylviane Dragacci

**Affiliations:** French Agency for Food, Environmental and Occupational Health Safety (Anses)–Food safety laboratory of Maisons-Alfort, European Union Reference Laboratory for Coagulase Positive Staphylococci, 23 avenue du Général de Gaulle, 94706 Maisons-Alfort, France; Email: annick.ostyn@anses.fr (A.O.); florence.guillier@anses.fr (F.G.); sabine.herbin@anses.fr (S.H.); anne-laure.prufer@anses.fr (A.-L.P.); sylviane.dragacci@anses.fr (S.D.)

**Keywords:** staphylococcal enterotoxin, food poisoning, enzyme immunoassay, molecular tools, mass spectrometry

## Abstract

Staphylococcal food poisoning is one of the most common food-borne diseases and results from the ingestion of staphylococcal enterotoxins (SEs) preformed in food by enterotoxigenic strains of *Staphylococcus aureus*. To date, more than 20 SEs have been described: SEA to SElV. All SEs have superantigenic activity whereas only a few have been proved to be emetic, representing a potential hazard for consumers. Characterization of staphylococcal food poisoning outbreaks (SFPOs) has considerably progressed compared to 80 years ago, when staphylococci were simply enumerated and only five enterotoxins were known for qualitative detection. Today, SFPOs can be characterized by a number of approaches, such as the identification of *S. aureus* biovars, PCR and RT-PCR methods to identify the *se* genes involved, immunodetection of specific SEs, and absolute quantification by mass spectrometry. An integrated gene-to-protein approach for characterizing staphylococcal food poisoning is advocated.

## 1. Coagulase-Positive Staphylococci and Staphylococcal Enterotoxins

### 1.1. Coagulase-Positive Staphylococci

*Staphylococcus* is a spherical, non-sporulating, non-motile bacterium (coccus) that, when observed under the microscope, occurs in pairs, short chains or grape-like clusters. These facultative aero-anaerobic bacteria are Gram- and catalase-positive. Staphylococci are ubiquitous in the environment and can be found in the air, dust, sewage, water, environmental surfaces, humans and animals.

To date, 50 species and subspecies of staphylococci have been described according to their potential to produce coagulase. Their classification thus distinguishes between coagulase-producing strains, designated as coagulase-positive staphylococci (CPS), and non-coagulase-producing strains, called coagulase-negative staphylococci (CNS). However, only CPS strains have been clearly implicated in food poisoning incidents. Among the seven described species belonging to the CPS group (Table 1), *S. aureus* subsp. *aureus* is the main causative agent described in staphylococcal food poisoning outbreaks (SFPOs). During processing and storage, temperatures outside the range of 7–48 °C prevent the growth of *S. aureus*. However, *S. aureus* subsp. *aureus* strains are usually very tolerant to NaCl and grow well in NaCl concentrations of up to 10%; growth is possible, although retarded, even in concentrations of up to 20%.

**Table 1:** Genus *Staphylococcus*: coagulase-positive species.

**Species**	**Main sources**	**Ref.**
*S. aureus* subsp. *aureus*	humans, animals	**[1]**
*S. aureus* subsp. *anaerobius*	sheep	**[2]**
*S. intermedius*	dog, horse, mink, pigeon	**[3]**
*S. hyicus*	pig, chicken	**[4]**
*S. delphini*	dolphin	**[5]**
*S. schleiferi* subsp. *coagulans*	dog (external ear)	**[6]**
*S. lutrae*	otter	**[7]**

### 1.2. Staphylococcal Enterotoxins

To date, 21 staphylococcal enterotoxins (SEs) and enterotoxin-like (SEl) types have been described (Table 2): enterotoxins A (SEA), B (SEB), C_1_ (SEC_1_), C_2_ (SEC_2_), C_3_ (SEC_3_), D (SED), E (SEE), G (SEG), H (SEH), I (SEI), J (SElJ)[8], K (SElK)[9], L (SElL), M (SElM), N (SElN), O (SElO)[10], P (SElP)[11], Q (SElQ)[12], R (SER)[13], S (SES), T (SET)[14], U (SElU)[15], and U2 and V, which are located in an open reading frame of the enterotoxin gene cluster *egc* that encodes enterotoxin-like proteins [16].

**Figure 1:**
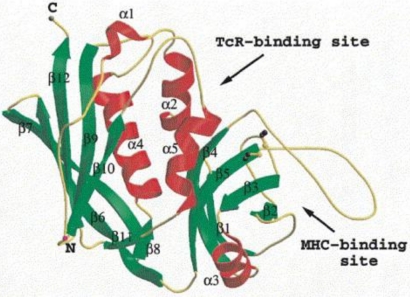
3D structure of SEB. Reproduced with permission from Elsevier [22].

Enterotoxin and enterotoxin-like proteins are globular, single polypeptides (Figure 1) with molecular weights ranging from 22 to 29 kDa. They can be encoded in prophages [17], plasmids [18] or chromosomal pathogenicity islands [19]. The currently known SEs form a group of serologically distinct, extracellular proteins that share important properties, namely: (1) the ability to cause emesis and gastroenteritis in primates; (2) superantigenicity through an unspecific activation of T lymphocytes followed by cytokine release and systemic shock [20]; (3) resistance to heat and to digestion by pepsin; and (4) structural similarities [21].

**Table 2:** Staphylococcal enterotoxin characteristics.

**Toxin type**	**Molecular weight (Da)**	**Genetic basis of SE**	**Superantigenic action**	**Emetic action**
SEA	27,100	Prophage	+	+
SEB	28,336	Chromosome, plasmid, pathogenicity island	+	+
SEC_1-2-3_	≈27,500	Plasmid	+	+
SED	26,360	Plasmid (pIB485)	+	+
SEE	26,425	Prophage	+	+
SEG	27,043	*enterotoxin gene cluster (egc)*, chromosome	+	+
SEH	25,210	Transposon	+	+
SEI	24,928	*egc*, chromosome	+	+
SElJ	28,565	Plasmid (pIB485)	+	nk
SEK	25,539	Pathogenicity island	+	nk
SElL	24,593	Pathogenicity island	+	−
SElM	24,842	*egc*, chromosome	+	nk
SElN	26,067	*egc*, chromosome	+	nk
SElO	26,777	*egc*, chromosome	+	nk
SElP	26,608	Prophage (Sa3n)	+	nk
SElQ	25,076	Pathogenicity island	+	−
SER	27,049	Plasmid (pIB485)	+	+
SES	26,217	Plasmid (pIB485)	+	+
SET	22,614	Plasmid (pIB485)	+	+
SElU	27,192	*egc*, chromosome	+	nk
SElU_2_	26,672	*egc*, chromosome	+	nk
SElV	24,997	*egc*, chromosome	+	nk

+: positive reaction; −: negative reaction; nk: not known.

## 2. SFPOs: Definition and Required Conditions for Their Occurrence (European Data)

Due to the previously enumerated properties of CPS and SEs, staphylococcal food poisoning (SFP) is one of the most common food-borne diseases and results from the ingestion of SEs preformed in food as these SEs are produced by enterotoxigenic strains of CPS, mainly *Staphylococcus aureus* [23]. 

The incubation period and severity of symptoms depend on the amount of enterotoxins ingested and the susceptibility of each individual. Initial symptoms—nausea followed by incoercible characteristic vomiting (in spurts)—appear within 30 min to 8 h (3 h on average) after ingestion of contaminated food. Other commonly described symptoms include abdominal pain, diarrhea, dizziness, shivering and general weakness sometimes associated with a moderate fever. In the most severe cases, headache, prostration and low blood pressure have been reported. In the majority of cases, recovery occurs within 24 to 48 h without specific treatment, while diarrhea and general weakness can last 24 h or longer. Death is rare, occurring primarily in those susceptible to dehydration (infants and elderly people) and in those affected by an underlying illness.

Five conditions are required to induce SFPOs: (1) a source containing enterotoxin-producing staphylococci: raw materials, healthy or infected carrier; (2) transfer of staphylococci from source to food: dirty food preparation tools due to poor hygiene practices; (3) food composition with favorable physico-chemical characteristics for *S. aureus* growth and toxinogenesis; (4) favorable temperature and sufficient time for bacterial growth and toxin production; and (5) ingestion of food containing sufficient amounts of toxin to provoke symptoms. 

Most SFPOs arise due to poor hygiene practices during processing [24], cooking or distributing the food product [25]. Staphylococci are commonly found in a wide variety of mammals and birds and transfer of *S. aureus* to food has two main sources: human carriage during food processing and dairy animals in case of mastitis. 

In Europe, the European Food Safety Authority [26] reported that, in 2008, bacterial toxins were involved in 525 out of 5332 notified food poisoning outbreaks (9.8%), ranking third in pathogenicity after *Salmonella spp.* (35.4%) and viruses (13.1%). Among bacterial toxins, SEs were involved in 291 out of the 525 notified food poisoning outbreaks (55.4%), or 5.5% of all notified outbreaks in 2008. 

## 3. Analytical Tools Used in SFPO Characterization: Pros and Cons

Diagnosis of SFP is generally confirmed by one of the following results: (1) the recovery of at least 10^5^ *S. aureus/*g from food remnants; (2) the detection of SEs in food remnants; and/or (3) the isolation of the same *S. aureus* strain from both patient and food remnants [27]. In some cases, confirmation of SFP is difficult because *S. aureus* is heat-sensitive, whereas SEs are not. Thus, in heat-treated food matrices, *S. aureus* may be eliminated without inactivating SEs. In such cases, it is not possible to characterize a food poisoning outbreak by enumerating CPS in food remnants or *a fortiori* detecting *se* genes in isolated strains. 

While *S. aureus* is classically enumerated using microbiological techniques with dedicated media such as Baird Parker or rabbit plasma fibrinogen agar media, three types of methods are usually performed to detect bacterial toxins in food: Bioassays, molecular biology and/or immunological techniques.

### 3.1. Bioassays

Bioassays are based on the capacity of an extract of the suspected food to induce symptoms such as vomiting, gastrointestinal symptoms in animals and/or superantigenic action in cell cultures. Historically, SEs have been detected based on their emetic activity in monkey-feeding and kitten‑intraperitoneal tests [28,29] and, more recently, using animal models such as house musk shrews *Suncus murinus* [14]. Symptoms of SFP appear if the dose ingested by the animals is above 200 ng, a considerably higher amount than those involved in human food poisoning [24,25,26,27,28,29,30]. Thus, this technique is not appropriate for characterizing SFPOs. More recently, a bioassay to detect the superantigenic activity of SEA has been developed [31]. This method uses SEA's superantigenic activity to induce in cytotoxic T lymphocytes a cytotoxic response against SEA-bound Raji cells. This test can only detect SEA at picomolar concentrations, and is thus of little interest for laboratories involved in official controls and SFP testings. 

In conclusion, in addition to the fact that the use of laboratory animals for testing is now restricted for ethical reasons, bioassays are not sensitive enough to ensure food safety for consumers. Thus, alternative methods for detecting SEs have been developed.

### 3.2. Molecular Tools

Molecular biology methods often involve the polymerase chain reaction (PCR). These methods usually detect genes encoding enterotoxins in strains of *S. aureus* isolated from contaminated foods. However, these methods have two major limitations: first, staphylococcal strains must be isolated from food, and second, the results inform as to the presence or absence of genes encoding SEs, but do not provide any information on the expression of these genes in food. This method therefore cannot be the sole method to detect SEs in food. However, the PCR approach is a specific, highly sensitive, and rapid method that can characterize the *S. aureus* strains involved in SFPOs, thereby providing highly valuable information. 

To improve SFP characterization, very recent efforts have been directed to determine which genes are involved in the biosynthesis of SEs. Following the huge SFP event which occurred in Japan in July 2000 (more than 13,000 people were intoxicated by powdered or liquid milk), Ikeda *et al.* [30] developed a PCR-based methodology whereby *sea*, *seg*, *seh* and *sei* genes could be detected in the incriminated powdered skim milk, although cultivable *S. aureus* were not recovered from the sample. Recently, to evaluate the toxic potential of strains isolated from SFPOs, various authors [32,33] have designed primers to perform PCR and reverse transcription PCR (RT-PCR) for *se* genes. These approaches demonstrate possible transcription of mRNA from those genes, but do not indicate whether those strains were able to produce detectable or poisonous levels of toxins in food. For example, Derzelle *et al.* [34] developed an RT-PCR-based procedure to determine the temporal expression of enterotoxin genes, including many of the newly discovered ones, in optimal growth conditions. PCR assays that can screen for 18 *se* genes have been developed and the distribution of these genes was examined on a panel of enterotoxigenic coagulase-positive staphylococci, including reference strains and isolates that have been collected from foods and SFPOs in France since the 1980s. A total of 28 strains displaying multiple enterotoxin genotypes were selected for further mRNA expression kinetics studies. 

More recently, Duquenne *et al.* [35] developed an efficient method to extract bacterial RNA accessible for RT-quantitative PCR (RT-qPCR) from cheese and adapted a simple, sensitive and reproducible, method for quantifying relative transcript levels to evaluate *S. aureus* enterotoxin gene expression during cheese manufacture. 

### 3.3. Immunological Tools

The third and most commonly used method for detecting SEs in food is based on the use of anti‑enterotoxin polyclonal or monoclonal antibodies. Commercially available kits have been developed according to two different principles: (1) enzyme immunoassay (EIA) comprising enzyme-linked immunosorbent assay (ELISA) and enzyme-linked fluorescent assay (ELFA); and (2) reverse passive latex agglutination (RPLA). 

It is widely recognized that the use of immunological methods to detect contaminants in food matrices is a difficult task, mainly due to the lack of specificity and sensitivity of the assay. Many drawbacks impair the development and use of these techniques for detecting SEs. First, highly purified toxins are needed to raise specific antibodies to develop an EIA; purified toxins are difficult and expensive to obtain. Moreover, and until very recently, only antibodies against SEA to SEE, SEG, SEH and SElQ have been available [36]. The ELISA test will not detect the other SEs, which partly explains some discrepancies that have arisen in the analysis of food extracts from SFPOs. Another drawback is the low specificity of some marketed kits, where false positives may occur depending on food components [37,38] as it is well known that some proteins, such as protein A, can interfere with binding to the Fc fragment (and, to a lesser extent, Fab fragments) in immunoglobins G from several animal species, such as mouse or rabbit, but not rat or goat. Other interferences are associated with endogenous enzymes, such as alkaline phosphatase or lactoperoxidase. 

Whatever the detection method used and due to the low amount of SEs present in food, it is crucial to concentrate the extract before performing detection assays. For this purpose, various methodologies have been tested [39,40,41]. Among them, only extraction followed by dialysis concentration has been approved by the EU to extract SEs from food [42].

However, up to now, after enumerating CPS strains, conclusive diagnosis of SFPs has been mainly based on demonstrating the presence of SEs in food using commercial EIA kits designed to detect SEA to SEE [43,44] or using a confirmatory in-house ELISA method [45] to differentiate and quantify these types of SEs. 

### 3.4. Chromatographic Methods for the Detection and Quantification of SEs

Due to drawbacks and the lack of available antibodies against the newly described SEs, other strategies based on physico-chemical techniques have been developed. Among these, mass spectrometry (MS) has recently emerged as an indispensable and suitable technique to analyze protein and peptide mixtures [46]. It is among the most sensitive techniques currently available because it provides specific, rapid and reliable analytical results. The development of two soft ionization methods, such as electrospray ionization (ESI) and matrix-assisted laser desorption/ionization (MALDI), and the use of appropriate mass analyzers have revolutionized the analysis of biomolecules. Given the wide range of methodologies available, a single MS technique cannot be used for all proteins [and all purposes]. The MS method thus requires the development of a series of techniques, individually suited for each particular case. 

In the case of food analysis, the situation is complex because the matrix can contain many proteins, lipids and many other molecular species that can interfere with the detection of the targeted toxin and may distort quantification. Sample preparation remains the critical step of the analysis. Several authors have tried to improve this step, by, for example, optimizing digestion parameters [47] or by adding a purification step [48]. The strategy of incorporating an isotopically labeled internal standard into the samples has also been developed. In the case of SE detection, some authors have developed MS tools to detect these toxins in culture supernatants and in spiked samples, such as water or apple juice. For example, Bernardo *et al.* [49] developed a MALDI-TOF method to detect *S. aureus* virulence factors such as enterotoxins and demonstrated that this technique was suitable for detecting SEs other than SEA to SEE in culture supernatants. In contrast, Callahan *et al.* [50] detected and quantified SEB using liquid chromatography coupled to ESI/MS detection in apple juice used as a model food matrix. In this study, enterotoxin types SEA and SEB were detected in spiked cheese. Recently, Brun *et al.* [51] developed an MS approach able to perform absolute quantification of SEA and TSST1 in spiked water or urine samples. To improve characterization and absolute quantification of SEs, this latter methodology was successfully used to carry out absolute quantification of SEA in a naturally contaminated cheese sample [52]. 

## 4. An Integrated Approach to Improve SFPO Characterization

To improve SFPO characterization, various techniques, such as immunological and molecular-based methodologies, have been integrated in the diagnosis strategy. The PCR approach is known to provide information on the presence or absence of genes encoding SEs, but not their expression. Nevertheless, PCR supplements classical methods, providing interesting additional data. In a study conducted on 178 *S. aureus* strains corresponding to 31 SFPOs isolated in France between 1981 and 2002, the results from a PCR assay revealed a satisfactory correlation (84%) with the results from immunoassay methods [53]. 

Due to the satisfactory results obtained, in 2005, the EU Reference Laboratory (EU-RL) for CPS, decided to use the PCR procedure to improve SFPO characterization. The diagnosis of SFPO essentially based on SEs has been significantly strengthened. For example, very recently, PCR on *se* genes has been used to demonstrate for the first time the presence of CPS strains carrying the *see* gene and able to produce the SEE in unpasteurized cheeses involved in six outbreaks in France [54]. 

To complete SFPO characterization, MS tools have been also used in combination with those presented above. Thus, an overall approach combining microbiology, molecular, immunological and quantitative mass spectrometry techniques was successfully used for investigations of SEs content in cheese [52] or in a dessert involving in food poisoning outbreaks [55].

## 5. Conclusions

To conclude, an overall approach combining classical microbiology to enumerate CPS strains coupled with immunological techniques, molecular biology and mass spectrometry-based methods offers an interesting alternative for assigning outbreaks to SEs. Thus, the development of standards to perform absolute quantification will continue. While the quantitative MS method overpasses specific technical limitations of existing ELISA methods for detecting and quantifying SEs, its throughput and cost per analysis compares unfavorably with ELISA. For this reason, when the MS-based method becomes available for all SEs involved in SFPOs it will not be employed for routine analysis, but only in special cases to confirm outbreaks due to SEs. 
